# Supplementary figures and images for: FAK downregulation suppresses stem-like properties and migration of human colorectal cancer cells
Source: PLoS One. 2023 Apr 21;18(4):e0284871. doi: 10.1371/journal.pone.0284871 (PMC10121060; doi:10.1371/journal.pone.0284871)

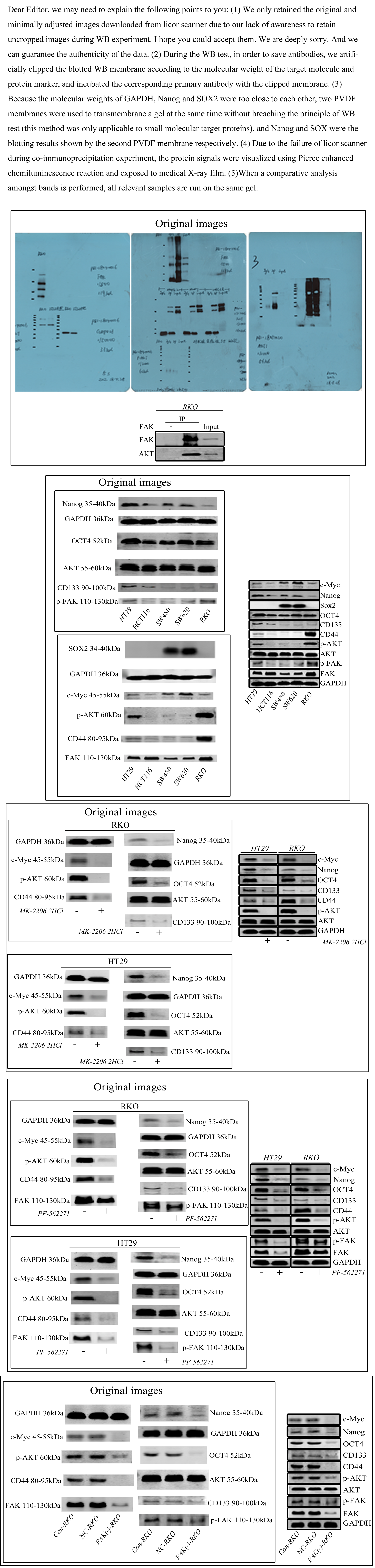

Supplement: S1 Raw images — (TIF) [file pone.0284871.s001.tif]
