# Supplementary material for: FAK downregulation suppresses stem-like properties and migration of human colorectal cancer cells
Source: PLoS One. 2023 Apr 21;18(4):e0284871. doi: 10.1371/journal.pone.0284871 (PMC10121060; doi:10.1371/journal.pone.0284871)
Supplement: Attachment — Submitted filename: Response to Reviewers.docx [file pone.0284871.s002.docx]

Response to Reviewers

**Reviewer #1**: Dear editor,

The manuscript entitled ‘FAK downregulation suppresses stem-like properties and migration of human colorectal cancer cells’ is interesting and novel in the area of cancer. CSCs and their contribution to therapy resistance and tumor progression is without a doubt of value, and the area still needs investigations.

1. The whole manuscript requires edition for grammar and writing errors in order to be a better draft for readers.

The grammar and writing errors of the manuscript have been revised and obtained a language certificate.

2. The sentence ‘cells protein was expression by western blotting analysis’ in the abstract section is wrong. Please rewrite the Abstract in order to be more direct and persuasive. Sentences like ‘Cell viability was measured by cell counting kit-8 and cells protein was expression by western blotting analysis and immunocytochemistry’, ‘Co-expression of FAK and AKT in colorectal cancer cells was assessed by co-immunoprecipitation’, and ‘Spheroid forming ability was evaluated and cell migration measured by wound healing assays’ must not be included in the abstract section. Instead, please refer to the key findings of the study.

We have rewritten the Abstract. I hope it could meet your requirement.

**ABSTRACT**

Focal adhesion kinase (FAK) is a cytoplasmic protein tyrosine kinase, which is overexpressed in colorectal cancer cells. FAK could be activated by phosphorylation to participate in the transduction of multiple signaling pathways and self-renewal of cancer stem cells. Whether downregulation of FAK inhibits the metastasis in colorectal cancer through weakening of stem cell-like properties and its mechanisms has yet to be established. CD44, CD133, c-Myc, Nanog, and OCT4 were known to mark colorectal cancer stem cell properties. In this study, AKT inhibitor (MK-2206 2HCl) or FAK inhibitor (PF-562271) decreased the expression of stem cell markers (Nanog, OCT4, CD133, CD44, c-Myc) and spheroid formation in colorectal cancer. Moreover, FAK and AKT protein was shown to interact verified by co-immunoprecipitation. Furthermore, downregulation of FAK, transfected Lenti-FAK-EGFP-miR to colorectal cancer cells, reduced p-AKT but not AKT and decreased the expression of stem cell markers and spheroid formation in colorectal cancer. In conclusion, we demonstrated that downregulation of FAK inhibited stem cell-like properties and migration in colorectal cancer cells partly due to altered modulation of AKT phosphorylation by FAK.

3. Introduction. The sentence ‘Both the EMT and the stem cell-like properties of tumor cells promote metastasis’ may pursue a wrong interpretation, as it has known EMT is a feature of cancer stemness not a separate event (10.1007/s12032-022-01801-0).

We have rewritten the sentence in Introduction.

During the initiation of metastasis, epithelial cancer cells undergo an epithelial-mesenchymal transition (EMT), and as a result become migratory and invasive mesenchymal-like cells while acquiring stem cell-like properties and therapy resistance ^[1, 2]^.

It was known EMT is a feature of stem cell-like properties in tumor ^[2]^.

4. AKT is primarily induced in CSCs through upregulation of the upstream Nanog (J. Clin. Investig. 122 (11) (2012) 4077–4093) or PI3K (Proc. Natl. Acad. Sci. USA 110 (17) (2013) 6829–6834). Where is the place here for FAK?

The AKT/Nanog pathway is critical for maintenance of sarcoma cancer stem cells and spheroid formation. Activation of AKT/β-catenin pathway maintains the growth of cancer stem cells and directly modulates the expression of Nanog and OCT4 in colorectal cancer (doi: 10.1038/s41389-020-00300-z.; doi:10.3892/ijmm.2021.4884). Inconsistently, some studies suggest that AKT is primarily induced in cancer stem cells through upregulation of the upstream Nanog (J. Clin. Investig. 122 (11) (2012) 4077–4093). A cross-regulation Maybe between AKT and Nanog, plays a significant role in maintains stem cell-like properties in tumor.

FAK inhibition can suppress ovarian cancer cells migration and invasion through inhibiting downstream PI3K/AKT signaling. (doi: 10.1002/cbin.10184.)

5. Introduction. Please indicate here that CSCs are equipped with highly efficient DNA repair and effective redox tolerance systems in order to promote therapy resistance (10.1016/j.biopha.2022.113906), and their number is increased outstandingly in cancers with cold immunity (10.1002/jbt.22708).

We have added the sentence in Introduction.

Cancer stem cells are equipped with highly efficient DNA repair and effective redox tolerance systems in order to promote therapy resistance ^[3]^, and their number is increased outstandingly in cancers with cold immunity ^[4]^.

**Reviewer #2**: The present study describes FAK regulation of stem cell-like properties of colon cancer cells.

FAK downregulation attenuates such stemness properties and cancer cell migration via AKT phosphorylation. FAK has been target of tumor-suppressor and downregulation of stem cells. The FAK science has well been documented to date. FAK in cytoplasmic region is present in cancer cells and p-AKT is upregulated in EMT, hepatoma and colon cancer metastasis. FAK/AKT/PI3K in EMT and stemness in cancer cells is also well known.

The present works are not justified for generalization, indicating a premature submission.

Downregulation of FAK inhibits the metastasis in colorectal cancer through weakening of stem cell-like properties and its mechanisms has yet to be established. Our works first demonstrated that downregulation of FAK inhibited stem cell-like properties and migration in colorectal cancer cells partly due to altered modulation of AKT phosphorylation by FAK.

1. More mechanistic explanation in FAK interaction with its partner molecules should be gel-shifted using immuno-precipitation.

In our research, FAK and AKT protein was shown to interact indeed verified by co-immunoprecipitation.

2. Several colorectal cancer cells have been analyzed in Fig.1 and etc. The genetic differences in p53 gene are characteristic and the FACK regulated is controlled by p53 and related genes. Therefore, the phenotype properties should be separately examined.

In Fig 1, we show the capacity of spheroid formation, cell migration and expression of FAK, p-FAK, AKT, p-AKT, CD44, CD133, OCT4, Nanog and c-Myc in HT29, HCT116, SW480, SW620 and RKO colorectal cancer cells. Expression of p-FAK and p-AKT was higher in HT29 and RKO. In consideration of FAK or AKT was manipulated by transfection experiments and specific inhibitor in subsequent experiments, HT29 and RKO cells were adopted. The results shown in Fig.1 could explain why HT29 and RKO were selected for the subsequent experiments, so the phenotype properties may be dispensable for separately examination.

3. PI3K/PTEn coregulation is missed

Some studies have reported that the FAK was involved in the regulation of cell mobility via activation of PI3K /AKT pathway, and associated phosphorylation of p85 subunits of tyrosine of PI3K in human cancer cells.

(doi:10.1038/nrc3792) ( doi:10.18632/oncotarget.6399)
