# Supplementary material for: FAK downregulation suppresses stem-like properties and migration of human colorectal cancer cells
Source: PLoS One. 2023 Apr 21;18(4):e0284871. doi: 10.1371/journal.pone.0284871 (PMC10121060; doi:10.1371/journal.pone.0284871)
Supplement: Attachment — Submitted filename: Response to Reviewers.docx [file pone.0284871.s003.docx]

Response to Reviewers

Dear reviewer,

Thank you for your letter and for the reviewer’ comments concerning our manuscript. Those comments are all valuable and very helpful for revising and improving our paper. We have studied comments carefully and have made correction which we hope meet with approval.

Reviewer #3:

1. Please carefully check the English grammar, which has many errors and typos.

We invited the high qualified and native English speakers at EditSprings again to modify the English grammar in our manuscript.

2. In Real time-PCR method, what is the thermal cycling conditions? How to calculate and analyze the level of gene expression? These should be specified in the method.

We added the thermal cycling conditions and the formula to calculate and analyze the level of gene expression in the method section.

**Real-time reverse transcription-polymerase chain reaction (RT-PCR) analysis**

Extraction of total RNA and reverse transcription was performed using extraction (TIANGEN, China) and reverse transcription (TaKaRa, Japan), as previously reported ^[^^13^^]^. FS universal SYBR green master (Roche, Germany) was used for cDNA synthesis via a fluorescence-based quantitative PCR method and real-time PCR System (ABI, USA). RT was performed using 2.0 µl 5X gDNA Eraser Buffer, 1.0 µl gDNA Eraser, 1 µg Total RNA and RNase Free dH2O to a total volume of 10 µl. The reaction conditions were 42˚C for 2 min, and rapidly cooled to 4˚C. Subsequently, 10.0 µl reaction solution, 1.0 µl PrimeScript RT Enzyme Mix 1, 1.0 µl RT Primer Mix, 4.0 µl 5X PrimeScript Buffer 2 and 4.0 µl RNase Free dH2O were used. The reaction conditions were 37˚C for 15 min, 85˚C for 5 sec, rapidly cooled to 4˚C and stored at ‑20˚C. A fluorescence‑based qPCR method was performed using 2 µl cDNA, 10 µl SYBR Green (Takara Bio, Inc.), 0.6 µl PCR forward primer (10 µM), 0.6 µl PCR reverse primer (10 µM) and 6.8 µl dH2O in a 20 µl PCR reaction volume. The RT‑qPCR reaction was run on a StepOne Real‑Time PCR system (Applied Biosystems; Thermo Fisher Scientific, Inc.). The cycling parameters were as follows: Denaturing at 95˚C for 30 sec, 40 cycles at denaturing at 95˚C for 5 sec, primer annealing at 60˚C for 34 sec and extension temperature at 95˚C for 15 sec; final extension at 60˚C for 1 min and final denaturing at 95˚C for 15 sec. Gene expression levels were determined using the 2^-ΔΔCq^ method. Primers for GAPDH and FAK were from TaKaRa, Japan. GAPDH, Forward: 5′-GCA CCG TCA AGG CTG AGA AC-3′, Reverse: 5′-TGG TGA AGA CGC CAG TGGA-3′; FAK, Forward: 5′-CAA CCA CCT GGG CCA GTA TTA TC-3′, Reverse: 5′-CCA TAG CAG GCC ACA TGC TTTA-3′.

3. In the results section, the authors have to explain the detail of each result more.

We made some changes in the results section. We hope it could meet your requirement.

**Results**

**Spheroid formation, cell migration and expression of FAK, p-FAK, AKT, p-AKT and stem cell marker proteins in HT29, HCT116, SW480, SW620 and RKO cells**

Spheroid formation, cell migration and expression of FAK, p-FAK, AKT, p-AKT and stem cell markers in HT29, HCT116, SW480, SW620 and RKO cells were assessed to aid the selection of two suitable colorectal cancer cell-lines for subsequent experiments. The above five colorectal cancer cell lines had differing capacities for spheroid formation (Fig 1A). FAK, p-FAK, AKT, p-AKT, CD44, CD133, OCT4, Nanog and c-Myc proteins were expressed in all five cell-lines, although Sox2 was not. Expression of p-FAK and p-AKT was higher in HT29 and RKO than in HCT116, SW480 and SW620 cells (Fig 1B). In consideration of FAK or AKT being manipulated by transfection experiments and specific inhibitors in subsequent experiments, HT29 and RKO cells were adopted.

**The effect of AKT inhibitor, MK-2206 2HCl, on survival of HT29 and RKO cells**

On treatment with 25 uM MK-2206 2HCl for 24 h, viability of HT29 and RKO cells, measured by CCK8, was not significantly reduced (Fig. 1C, p>0.05). At this time, MK-2206 2HCl could exert maximum efficacy in inhibiting target protein expression with minimal toxicity to the survival of HT29 or RKO cells.

**MK-2206 2HCl inhibited stem-like properties and migration of colorectal cancer cells**.

Cell immunocytochemistry analysis showed that expression of p-AKT, CD44 and c-Myc proteins was decreased by 24 h treatment with 25 uM MK-2206 2HCl for RKO cells (Fig 2A, p<0.05). In order to further clarify the impact of MK-2206 2HCl on the proteins expression of AKT, p-AKT and cancer stem cell markers, the proteins of HT29 and RKO cells were also detected by western blotting. Treatment with 25 uM MK-2206 2HCl for 24 h reduced expression of p-AKT, CD44, CD133, OCT4, Nanog and c-Myc (p<0.05) while that of AKT was unchanged (p＞0.05) in HT29 and RKO cells (Fig 2B). In the meantime, the spheroid forming ability was reduced (Fig 2C, p<0.05), as was migration distance (Fig 2D, p<0.05), indicating loss of migratory capacity. It has been shown that p-AKT regulated the expression of cancer stem cell markers previously ^[^^16^^]^. The above findings show that MK-2206 2HCl reduced stem cell-like properties and migration of colorectal cancer cells via suppression of p-AKT expression.

**The effect of FAK inhibitor, PF-562271, on survival of HT29 and RKO cells.** Treatment with 5 uM PF-562271 for 24 h did not reduce HT29 and RKO cell viability as measured by CCK8 (Fig 1D, p＞0.05). At this time, PF-562271 could exert maximum efficacy in inhibiting target protein expression with minimal toxicity to the survival of HT29 or RKO cells.

**PF-562271 inhibited stem-like properties and migration of colorectal cancer cells though suppression of p-AKT expression**

The RKO cells were treated with 5 uM PF-562271 for 24 h decreased p-FAK, p-AKT, CD44 and c-Myc expression by cell immunocytochemical analysis (Fig 3A, p<0.05) and expression of FAK, p-FAK, p-AKT, CD44, CD133, OCT4, Nanog and c-Myc by western blotting (p<0.05) in both HT29 and RKO cells while AKT was unchanged (p>0.05) (Fig 3B). In the meantime, HT29 and RKO spheroid formation was reduced (Fig 3C, p<0.05), as was migration distance (Fig 3D, p<0.05). The above findings supported that PF-562271 inhibited stem cell-like properties and migration of colorectal cancer cells perhaps via suppression of the FAK/p-FAK/p-AKT axis.

**Downregulation of FAK suppressed p-AKT expression and inhibited stem cell-like properties and migration of colorectal cancer cells**

RKO cells were stably transfected with Lenti-FAK-EGFP-miR to reduce FAK expression and FAK mRNA was successfully suppressed (Fig 4A, p<0.05). Expression of FAK, p-FAK, p-AKT, CD44, CD133, OCT4, Nanog and c-Myc decreased (p<0.05) when FAK was downregulated and no change was seen in AKT expression (p>0.05) (Fig 4B). RKO cells after FAK knockdown [FAK(-)-RKO] showed reduced spheroid formation compared with transfected negative control (NC) and control (Con) RKO cells (Fig 4D, p<0.05). Moreover, migrational potency was significantly suppressed by FAK knockdown (Fig 4E, p<0.05).

**FAK regulated AKT phosphorylation in colorectal cancer cells**

FAK and AKT proteins were shown to interact by co-immunoprecipitation in RKO cells (Fig 4C). The RKO cells were treated with 5 uM PF-562271 for 24 h reduced p-AKT expression (p<0.05) but not that of AKT (p>0.05) (Fig 3B). Moreover, FAK knockdown in RKO cells reduced p-AKT expression (p<0.05) but not that of AKT (p>0.05) (Fig 4B). FAK appears to regulate AKT phosphorylation in colorectal cancer cells. In summary, the above findings show that the downregulation of FAK suppressed stem cell-like properties and migration of colorectal cancer cells partly by FAK modulation of AKT phosphorylation.

4. All images of all figures have not the scale bar, it needs to show in the image and also specify in the figure legends.

We added the scale bar in all images of all figures. I hope it could meet your requirement.

**Fig 1.** **Spheroid formation, cell migration, protein expression in colorectal cancer cells and the effect of MK-2206 2HCl and PF-562271 on survival of cells.** (A) Harvested cells were seeded under nonadherent culture conditions for spheroid formation (magnification, ×40). Numbers of spheroids were quantified after 7 days. (B) Protein levels assayed by western blotting. GAPDH was used as an internal control. (C) Viability of HT29 and RKO cells after treatment with MK-2206 2HCl. (D) Viability of HT29 and RKO cells after treatment with PF-562271. *p<0.05 compared with control group (0 µM).

**Fig 2. MK-2206 2HCl inhibited stem-like properties and migration of HT29 and RKO cells.** (A) Protein levels were assayed by immunocytochemistry analysis (magnification, ×200). (B) Protein levels were assayed by western blotting. GAPDH was used as an internal control. (C) The harvested cells were seeded under nonadherent culture conditions for spheroid formation (magnification, ×40). Number of spheroids was quantified after 7 days. (D) Migratory potency of cells demonstrated by wound healing assays (magnification, ×100) and analysis. (*p<0.05)

**Fig 3.** **PF-562271** **inhibited stem-like properties and migration of HT29 and RKO cells.** (A) Protein levels were assayed by immunocytochemistry analysis (magnification, ×200). (B) Protein levels were assayed by western blotting. GAPDH was used as an internal control. (C) Harvested cells were seeded under nonadherent culture conditions for spheroid formation (magnification, ×40). Numbers of spheroids were quantified after 7 days. (D) Migratory potency of cells demonstrated by wound healing assays (magnification, ×100) and analysis. (*p<0.05)

**Fig 4**. **Downregulation of FAK inhibited stem cell-like properties and migration of colorectal cancer cells through reducing AKT phosphorylation.** (A) Fluorescence microscopy of transfected and un-transfected RKO cells (magnification, ×100). Bar graphs illustrating RT-PCR analysis of FAK mRNA. GAPDH was used as a reference and the Con-RKO was set to 1. (B) Protein levels were assayed by western blotting. GAPDH was used as an internal control. (C) Interaction of FAK and AKT proteins in colorectal cancer cells shown by co-immunoprecipitation. (D) Harvested cells were seeded under nonadherent culture conditions for spheroid formation (magnification, ×40). Numbers of spheroids were quantified after 7 days. (E) Migratory potency of cells demonstrated by wound healing assays (magnification, ×100) and analysis. (*p<0.05)

5. The authors need to link and interpret the data in the results and discussion about the two different cell lines, HT29 and RKO.

In the discussion section, we have linked and interpreted the data of the results. HT29 and RKO are all colorectal cancer cell lines, so we did not discuss them separately.

**Discussion**

Cancer stem cells represent a population of cells within a tumor with highly tumorigenic properties and may be identified by the expression of cancer stem cell markers. Cancer stem-like cells make a major contribution to metastasis and are known to exist in a variety of solid tumors, including colorectal cancer ^[^^19^^,^ ^20^^]^. Targeting cancer stem-like cells appears to be a promising approach for the elimination of cancer recurrence and improvement of clinical outcomes ^[^^21^^]^.

AKT is thought to be involved in stem cell survival. Activation of AKT, such as the action of PI3K leading to phosphorylation of AKT to p-AKT, increased the number of neural stem cells ^[^^22^^,^ ^23^^]^. The AKT/Nanog pathway is critical for the maintenance of sarcoma cancer stem cells and spheroid formation. Activation of the AKT/β-catenin pathway maintains the growth of cancer stem cells and directly modulates the expression of Nanog and OCT4 in colorectal cancer ^[^^24^^,^ ^25^^]^. Inconsistently, some studies suggest that AKT is primarily induced in cancer stem cells through upregulation of the upstream Nanog ^[^^26^^]^. A cross-regulation between AKT and Nanog may play a significant role in maintaining stem cell-like properties in tumors. In addition, AKT activation underpinned the stemness of colorectal cancer stem cells and CD133 expression changed when AKT was inhibited ^[^^23^^]^. Increased CD44 expression in colorectal cancer cells was shown to be linked to increased AKT expression by the superimposition of targeted proteomic analysis on transcriptomic analysis ^[^^27^^,^ ^28^^]^. C-Myc also was a downstream molecule in the AKT pathway and siRNA knockdown of c-Myc effectively inhibited cancer stem cell-like features ^[^^29^^]^. In the current study, low expression of p-AKT was associated with decreased expression of stem cell markers, Nanog, OCT4, CD133, CD44 and c-Myc, in RKO and HT29 cells. Spheroid formation was suppressed by AKT inactivation. These data indicated colorectal cancer cells losed stem cell-like properties partly by inactivation of AKT with reduced phosphorylation of AKT.

FAK, which is implicated in tumor growth and metastasis, traffic to and is present in the nucleus, affecting gene regulation in a kinase-independent manner. The anti-tumor activity of FAK inhibitors has been used to reinforce traditional cytotoxic chemotherapy and immunotherapy in the form of combination treatments. PI3K activates AKT by phosphorylating serine 473 (Ser473) and the FAK/PI3K/AKT pathway is known to regulate cell motility. FAK was involved in the regulation of cell mobility via activation of the PI3K /AKT pathway, and associated phosphorylation of p85 subunits of tyrosine of PI3K in human cancer cells ^[^^28^^,^ ^30^^].^ FAK and AKT proteins were shown to interact verified by co-immunoprecipitation and inhibition of FAK reduced p-AKT expression in this study. These data suggest a role for FAK in the regulation of AKT phosphorylation in colorectal cancer cells. FAK inhibition decreases cancer stem-like properties. FAK is implicated in modulating the resistant and aggressive phenotype of cancer stem cells ^[^^31^^]^. The present study shows that FAK inhibitor decreased the expression of p-AKT and of stemness-related proteins, such as Nanog, OCT4, CD133, CD44 and c-Myc, in RKO and HT29 cells. When FAK expression of the transfected RKO cells was suppressed, levels of p-AKT and stemness markers were also decreased. FAK inhibition suppressed spheroid formation and migration of colorectal cancer cells.

In conclusion, the current study demonstrated that downregulation of FAK inhibited stem cell-like properties and migration of colorectal cancer cells partly due to altered modulation of AKT phosphorylation by FAK. Besides, pluripotency-associated factor Nanog expression was significantly attenuated by ERK knockdown ^[^^32^^]^. FAK regulates cancer cell migration and stemness via ERK/Nanog pathway ^[^^33^^]^. FAK is a potential tumor-suppressor target for colorectal cancer treatment, leading to reduced stem cells. Colorectal cancer stem-like cells are considered to be a primary cause of tumor recurrence. Elimination of cells with stem-like properties would make a significant contribution to improving clinical outcomes in patients with colorectal cancer.We sincerely hope that this revised manuscript has addressed all your comments and suggestions. We appreciated for reviewers’ warm work earnestly, and hope that the correction will meet with approval. Once again, thank you very much for your comments and suggestions.

We sincerely hope that this revised manuscript has addressed all your comments and suggestions. We appreciated for reviewer’ warm work earnestly, and hope that the correction will meet with approval. Once again, thank you very much for your comments and suggestions.
