# Supplementary material for: FAK downregulation suppresses stem-like properties and migration of human colorectal cancer cells
Source: PLoS One. 2023 Apr 21;18(4):e0284871. doi: 10.1371/journal.pone.0284871 (PMC10121060; doi:10.1371/journal.pone.0284871)
Supplement: Attachment — Submitted filename: Response to Reviewers.docx [file pone.0284871.s004.docx]

Response to Reviewer

Dear reviewer,

Thank you for your letter and for the reviewer’ comments concerning our manuscript. This comment is valuable and very helpful for revising and improving our paper. We have studied the comment carefully and have made correction which we hope meet with approval.

Reviewer #3:

All images of all figures have already added the scale bar, but in the figure legends are not correct. Authors only wrote the word "(magnification, ×40)", so it should be written "(magnification, ×40; scale bar 20 μm)". Please edit and rewrite them all of the figure legends.

We edit and rewrite the figure legends as your suggestion. I hope it could meet your requirement.

**Fig 1.** **Spheroid formation, cell migration, proteinexpression in colorectal cancer cells and the effect of MK-2206 2HCl and PF-562271 on survival of cells.** (A) Harvested cells were seeded under nonadherent culture conditions for spheroid formation (magnification, ×40；scale bar 20 μm). Numbers of spheroids were quantified after 7 days. (B) Protein levels assayed by western blotting. GAPDH was used as an internal control. (C) Viability of HT29 and RKO cells after treatment with MK-2206 2HCl. (D) Viability of HT29 and RKO cells after treatment with PF-562271. *p<0.05 compared with control group (0 µM).

**Fig 2. MK-2206 2HCl inhibited stem-like properties and migration of HT29 and RKO cells.** (A) Protein levels were assayed by immunocytochemistry analysis (magnification, ×200；scale bar 50 μm). (B) Protein levels were assayed by western blotting. GAPDH was used as an internal control. (C) The harvested cells were seeded under nonadherent culture conditions for spheroid formation (magnification, ×40；scale bar 20 μm). Number of spheroids was quantified after 7 days. (D) Migratory potency of cells demonstrated by wound healing assays (magnification, ×100；scale bar 100 μm) and analysis. (*p<0.05)

**Fig 3.** **PF-562271** **inhibited stem-like properties and migration of HT29 and RKO cells.** (A) Protein levels were assayed by immunocytochemistry analysis (magnification, ×200；scale bar 50 μm). (B) Protein levels were assayed by western blotting. GAPDH was used as an internal control. (C) Harvested cells were seeded under nonadherent culture conditions for spheroid formation (magnification, ×40；scale bar 20 μm). Numbers of spheroids were quantified after 7 days. (D) Migratory potency of cells demonstrated by wound healing assays (magnification, ×100；scale bar 100 μm) and analysis. (*p<0.05)

**Fig 4**. **Downregulation of FAK inhibited stem cell-like properties and migration of colorectal cancer cells through reducing AKT phosphorylation.** (A) Fluorescence microscopy of transfected and un-transfected RKO cells (magnification, ×100；scale bar 100 μm). Bar graphs illustrating RT-PCR analysis of FAK mRNA. GAPDH was used as a reference and the Con-RKO was set to 1. (B) Protein levels were assayed by western blotting. GAPDH was used as an internal control. (C) Interaction of FAK and AKT proteins in colorectal cancer cells shown by co-immunoprecipitation. (D) Harvested cells were seeded under nonadherent culture conditions for spheroid formation (magnification, ×40；scale bar 20 μm). Numbers of spheroids were quantified after 7 days. (E) Migratory potency of cells demonstrated by wound healing assays (magnification, ×100；scale bar 100 μm) and analysis. (*p<0.05)

We sincerely hope that this revised manuscript has addressed all your comments and suggestions. We appreciated for reviewer’ warm work earnestly, and hope that the correction will meet with approval. Once again, thank you very much for your comments and suggestions.
